# Risk of acute kidney injury in hospitalized patients with inflammatory bowel disease: a systematic review and meta-analysis

**DOI:** 10.1093/jcag/gwag004

**Published:** 2026-02-13

**Authors:** Bachviet Nguyen, Stephanie Quon, Elias Hazan, Megan Borkum, Sarvee Moosavi

**Affiliations:** Department of Medicine, University of British Columbia, Vancouver, British Columbia, V5Z 1M9, Canada; Department of Medicine, University of British Columbia, Vancouver, British Columbia, V5Z 1M9, Canada; Division of Gastroenterology, Department of Medicine, University of British Columbia, Vancouver, British Columbia, V5Z 1M9, Canada; Division of Nephrology, Department of Medicine, University of British Columbia, Vancouver, British Columbia, V5Z 1M9, Canada; Division of Gastroenterology, Department of Medicine, University of British Columbia, Vancouver, British Columbia, V5Z 1M9, Canada

**Keywords:** inflammatory bowel disease, acute kidney injury, systematic review, meta-analysis

## Abstract

**Objectives:**

Inflammatory bowel disease (IBD) is associated with a range of extraintestinal manifestations, including renal complications. While chronic kidney disease in IBD is well described, the risk of acute kidney injury (AKI) remains less well quantified. We aimed to evaluate the risk of AKI among hospitalized patients with IBD compared to non-IBD populations, and to assess this risk across clinical subgroups.

**Methods:**

We conducted a systematic review and meta-analysis in accordance with PRISMA (Preferred Reporting Items for Systematic Reviews and Meta-Analyses) guidelines and registered the protocol with PROSPERO. A comprehensive search of PubMed, MEDLINE, Embase, Scopus, and Cochrane CENTRAL was conducted from inception to June 2025. Eligible studies included cohort, case-control, and randomized control trials reporting on AKI outcomes in IBD versus non-IBD comparators. Meta-analyses were performed using random-effects models. Subgroup analyses were conducted by surgical status, infection, acute coronary syndrome, and general hospitalization.

**Results:**

Seventeen retrospective cohort studies involving 20 127 976 patients (140 482 with IBD) were included. IBD was associated with significantly increased odds of AKI (pooled odds ratio [OR]: 1.87; 95% confidence interval [CI], 1.53-2.29). The association was especially prominent in surgical patients (OR: 2.17; 95% CI, 1.73-2.73), including orthopedic (OR: 2.22; 95% CI, 1.50-3.30) and spinal (OR: 2.15; 95% CI, 1.66-2.78) subgroups. Associations in acute coronary syndrome and infection subgroups were less consistent. ROBINS-E (Risk Of Bias In Non-randomized Studies—of Exposures) assessments revealed a moderate risk of bias.

**Conclusions:**

A diagnosis of IBD is potentially associated with the development of AKI, particularly in surgical settings. Routine renal monitoring could be considered, especially during hospitalizations and perioperative care.

## Introduction

Inflammatory bowel disease (IBD), which includes Crohn’s disease (CD) and ulcerative colitis (UC), is a chronic inflammatory condition primarily affecting the gastrointestinal tract but frequently accompanied by extraintestinal manifestations, including renal complications.^1^^,^^2^ Nephrolithiasis is the most common complication, particularly in CD and often linked to active disease or prior intestinal resection. IBD is also associated with a wide range of renal pathologies, including tubulointerstitial nephritis (TIN), IgA nephropathy, amyloidosis, and glomerulonephritis.^3^^,^^4^ Large-scale epidemiological studies show higher rates of both acute kidney injury (AKI) and chronic kidney disease (CKD) in IBD patients compared to the general population, with adjusted odds ratios (ORs) for CKD up to 1.59.^5^^,^^6^

Acute kidney injury has been potentially linked to IBD, with possible mechanisms including diarrhea-related volume depletion and systemic inflammation, and potentially nephrotoxic medications such as 5-aminosalicylic acid (5-ASA), although the nephrotoxicity of 5-ASA is controversial.^1^^,^^4^^,^^7^^,^^8^ TIN is the most frequently reported histological lesion in IBD-associated AKI. Although often attributed to 5-ASA exposure, it may also occur in drug-naive ­patients, suggesting an inflammatory or autoimmune mechanism.^8^^,^^9^ The risk of AKI is increased in both CD and UC, independent of traditional renal risk factors.^10^

The risk for AKI among IBD patients seems highest in those with active disease, those requiring hospitalization, and ­individuals with a history of surgical interventions such as colectomy or long-term stoma use.^10^^,^^11^ The inpatient setting, in particular, has been associated with disproportionately high AKI rates, especially among older adults and those with comorbidities.^12^

Findings across individual studies vary, and no systematic review or meta-analysis has comprehensively quantified the risk of AKI in individuals with IBD to date. We aimed to address this gap by synthesizing available literature to determine the extent of AKI risk in patients with IBD, evaluate the role of disease phenotype and medication exposure, and inform targeted strategies for prevention and monitoring.

## Methods

### Search strategy

This systematic review and meta-analysis adhered to the PRISMA (Preferred Reporting Items for Systematic Reviews and Meta-Analyses) guidelines^13^ and MOOSE (Meta-analysis of Observational Studies in Epidemiology) guidelines.^14^ The study was registered with PROSPERO (CRD420251091101) before data extraction.

A comprehensive search of PubMed, MEDLINE, Embase, Scopus, and Cochrane CENTRAL was conducted from inception to July 9, 2025. A medical librarian assisted in optimizing the search strategy, which included both keywords and MeSH terms related to “inflammatory bowel disease,” “Crohn’s disease,” “ulcerative ­colitis,” “acute kidney injury,” “renal impairment,” and “nephrotoxicity.” Reference lists of included studies and relevant reviews were manually searched to identify additional eligible studies. Two independent reviewers (B.N. and S.Q.) screened titles and abstracts for eligibility using Covidence (Covidence systematic review software, Veritas Health Innovation, Melbourne, Australia).

Studies were included if the population consisted of hospitalized patients with IBD (CD or UC), the comparator group included non-IBD controls or baseline populations without IBD, outcomes reported included the incidence or comparative risk of AKI defined by standardized criteria or author-defined diagnosis. The study design was a retrospective observational analysis of hospitalization events (eg, database-based cross-sectional or cohort analyses of discrete inpatient encounters), case-control study, or randomized controlled trial. Only studies published in English were included. Exclusion criteria included case series with fewer than 10 IBD patients, studies without a comparator group, studies reporting only CKD or end-stage renal disease without AKI outcomes, conference abstracts, reviews, letters, or editorials, and studies without an available full text.

Two independent reviewers (B.N. and S.Q.) conducted the full-text review (Appendix B, Figure S1). Any discrepancies were resolved through consultation with a third reviewer (E.H.). Extracted data included author and publication year, country, study design, data time range, data source, definitions of IBD and AKI, type of IBD (eg, CD, UC, or unspecified), subgroup analyses (eg, surgical, infection, acute coronary syndrome), reason for hospitalization, total population size, number of IBD and non-IBD patients, and reported effect measures including ORs, and confidence intervals (CIs).

The PROSPERO-registered protocol prespecified subgroup analyses by age, sex, baseline comorbidity burden, and IBD ­subtype. However, these analyses were not feasible because the included studies did not provide stratified effect estimates or sufficient raw data to support these comparisons.

### Risk of bias assessment

Risk of bias for cohort and case-control studies was assessed using the Risk Of Bias In Non-randomized Studies—of Exposures (ROBINS-E) tool,^15^ and visualized using the *robvis* traffic light plot tool.^16^

### Statistical methods

All meta-analyses were conducted using R version 4.3.1 (R Foundation for Statistical Computing, Vienna, Austria) with the *metafor* package (version 4.8.0). Effect sizes were reported as ORs with corresponding 95% CIs, as extracted from each study. Where studies reported both crude and adjusted effect estimates, we preferentially extracted the most fully adjusted OR to maximize comparability between IBD and non-IBD cohorts. When only raw event counts or unadjusted estimates were available, we calculated or used unadjusted ORs. For studies that reported only point estimates and CIs, the standard errors were calculated.

A random-effects model was used for all analyses. Between-study variance (τ^2^) was estimated using the restricted maximum likelihood approach. Heterogeneity was quantified ­using Cochran’s *Q* test and the *I*^2^ statistic, which describes the ­percentage of total variation due to between-study heterogeneity. The degree of heterogeneity was interpreted based on Cochrane thresholds (*I*^2^ was 25% or below, moderate when between 26% and 50%, substantial when between 51% and 75%, and considerable when above 75%).^17^

Subgroup analyses were conducted based on clinical context, including surgical setting (overall, orthopedic, spinal), ­nonsurgical populations, infection, acute coronary syndrome, and studies including any reason for hospitalization. Separate random-effects models were fitted for each subgroup.

Forest plots were generated to visually display the effect estimates and 95% CIs for each study and the pooled estimates. All statistical tests were 2-sided, and *P *< .05 was considered statistically significant. Publication bias was evaluated for the overall OR analyses through visual inspection of funnel plots and Egger’s regression test. Due to the small sample size (*k* < 10), funnel plots and Egger’s tests were not conducted for all analyses.

## Results

### Descriptive overview

A total of 17 retrospective observational studies from 6 countries were included, with data spanning from 2005 to 2022 (Table 1).^6^^,^^10^^,^^18–32^ A total of 20 127 976 patients were included, with 140 482 patients with IBD. Most studies were conducted in the United States (*n* = 13). Sample sizes ranged from 122 to over 2.3 million patients. All studies relied on large-scale administrative or clinical databases (eg, National Inpatient Sample). Thirteen studies included surgical patients, while 4 focused on nonsurgical cohorts. All included patients with IBD, either exclusively or as a defined subgroup within broader cohorts. All studies identified AKI using diagnostic codes from the International Classification of Diseases (ICD) system, and AKI was defined based on ICD-9 codes (eg, 584.x) or ICD-10 codes (eg, N17.x). All included studies evaluated hospitalized patients only.

**Table 1:** Overview of included studies in the systematic review (*n* = 17).

**Author (year)**	Country	Study design	Time range	Data source	Reason for hospitalization	Surgical vs. nonsurgical	Total number of patients	Total number of patients with IBD	Adjustment covariates
**Pemmasani et al. (2021)^25^**	USA	Retrospective cohort study	2005-2015	National Inpatient Sample	Acute coronary syndrome	Nonsurgical	6 896 635	24 220	Age, demographics, CV risk factors, comorbidities, ACS presentation type, in-hospital complications^b^ ^c^
**Antia et al. (2024)^26^**	USA	Retrospective cohort study	2016-2020	National Inpatient Sample	Acute coronary syndrome	Nonsurgical	2 367 475	11 837	Age, sex, race, comorbidities, baseline oxygen use, hospital characteristics
**Yang et al. (2024)^10^**	Sweden	Retrospective cohort study	2006-2019	SCREAM Project	Any reason for hospitalization	N/A^a^	1 683 644	5483	Age, sex, baseline GFR, comorbidities, medications^d^
**Liu et al. (2023)^6^**	UK	Retrospective cohort study	2006-2010	UK Biobank	Any reason for hospitalization	N/A^a^	417 338	4201	Age, sex, race
**Saha et al. (2024)^19^**	USA	Retrospective cohort study	2014	HCUP-NIS	Any reason for hospitalization	N/A^a^	5 575 874	57 121	Age, sex, race/ethnicity, hospital type, hospital region, comorbidities tobacco use, contrast exposure
**Aldiabat et al. (2024)^32^**	USA	Retrospective cohort study	2020	National Inpatient Sample	Infection	Nonsurgical	1 050 043	5748	Age, gender, race, income, comorbidities alcohol use, long-term steroid use, hospital characteristics
**Ukash et al. (2022)^18^**	Israel	Retrospective cohort study	2012-2018	Single Centre	Infection	Nonsurgical	21 808	122	Age, sex, IBD disease extent, prior IBD surgery, IBD medications comorbidities, prior hospitalization^e^
**Bazerbachi et al. (2018)^20^**	USA	Retrospective cohort study	2011-2013	Nation-wide Inpatient Sample	Bariatric surgery	Surgery	314 864	790	Age, sex, race, comorbidities
**Kim et al. (2022)^27^**	USA	Retrospective cohort study	2009-2013	Statewide Planning and Research Cooperative System	Orthopedic surgery	Surgery	89 134	244	Age, sex, race, insurance type, comorbidities, CD status^f^
**Silvestre et al. (2025)^28^**	USA	Retrospective case-control study	2010-2020	National Readmission Database	Orthopedic surgery	Surgery	4935	1249	Age, sex, income quartile, insurance type, smoking, alcohol use, chronic corticosteroid use, comorbidities
**Sun et al. (2024)^29^**	USA	Retrospective cohort study	2016-2019	Nationwide Inpatient Sample	Orthopedic surgery	Surgery	558 371	1461	No adjustment
**Hinkle et al. (2025)^30^**	USA	Retrospective cohort study	2016-2019	Nationwide Inpatient Sample	Orthopedic surgery	Surgery	367 390	1171	No adjustment
**Magruder et al. (2023)^31^**	USA	Retrospective cohort study	2010-2020	PearlDiver Claims Database	Orthopedic surgery	Surgery	66 146	11 025	Age, sex, comorbidities
**Seddio et al. (2024)^22^**	USA	Retrospective cohort w/matching	2010-2022	National Insurance Claims Database	Spinal surgery	Surgery	306 404	4392	Age, sex, comorbidities
**Elali et al. (2025)^21^**	USA	Retrospective cohort study	2010-2020	Mariner or Medicare Claims	Spinal surgery	Surgery	20 640	3446	Age, sex, comorbidities
**Zhang et al.(2024)^23^**	China	Retrospective cohort study	2010-2020	PearlDiver Patient Records	Spinal surgery	Surgery	36 500	6134	Age, gender, comorbidities
**Yan et al.(2025)^24^**	USA	Retrospective cohort study	2010-2019	Nationwide Inpatient Sample	Spinal surgery	Surgery	350 775	1838	Age, gender, race, insurance type, hospital bed size, hospital teaching status, hospital location, elective admission, comorbidities, and perioperative complications.

a N/A: non-applicable

b CV: cardiovascular

c ACS: acute coronary syndrome

d GFR: glomerular filtration rate

e IBD: inflammatory bowel disease

f CD: Crohn's disease

### Pooled ORs—all cause hospitalizations

**Figure 1:**
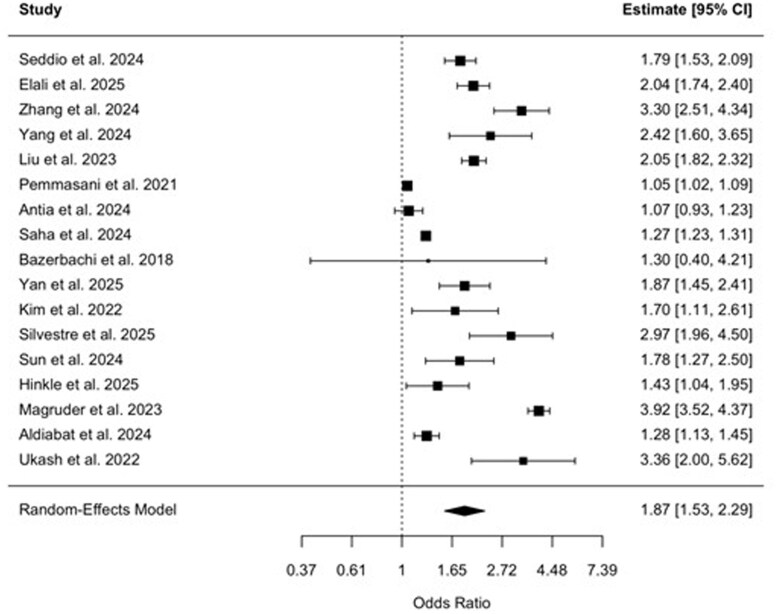
Forest plot of 17 studies assessing the odds of acute kidney injury in patients with inflammatory bowel disease. The pooled odds ratio was 1.87 (95% CI, 1.53-2.29) using a random-effects model.

**Figure 2:**
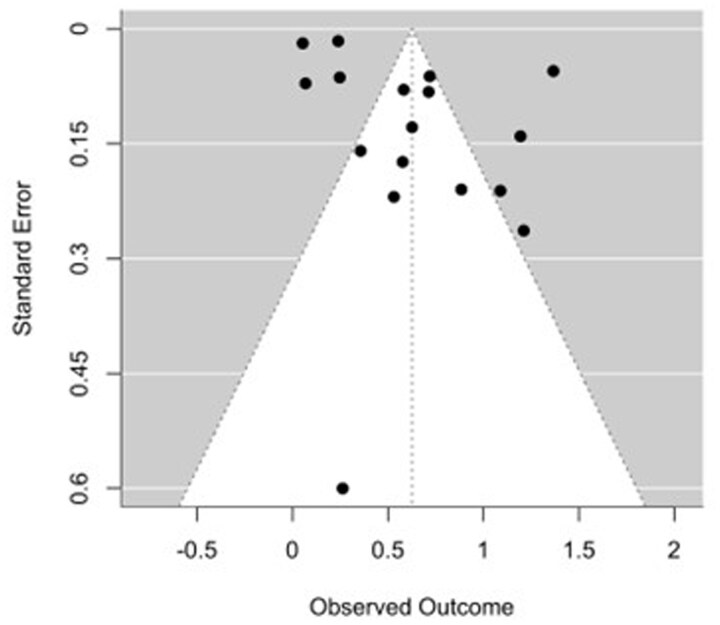
Funnel plot of included studies for assessment of publication bias. Egger’s test was nonsignificant (*P* = .325), suggesting no major evidence of small-study effects or asymmetry.

The 17 cohort studies^6^^,^^10^^,^^18–32^ included in the meta-analysis assessed the association between IBD and AKI across all hospitalizations. The pooled OR for AKI among patients with IBD was 1.87 (95% CI, 1.53-2.29, *P *< .0001), demonstrating a statistically significant risk compared to non-IBD populations (Figure 1). Heterogeneity was considerable, with an *I*^2^ = 98.1% and τ^2^ = 0.154 (*Q*(16) = 725.57, *P *< .0001). Visual inspection of the funnel plot did not reveal marked asymmetry, and Egger’s regression test was not statistically significant (*z *= 0.985, *P* = .325) (Figure 2). A sensitivity analysis of studies published between 2023 and 2025 was also completed (Appendix B, Figure S2), which found a pooled OR of 1.93 (95% CI, 1.55-2.39; *P *< .0001) with considerable heterogeneity (*I*^2^ = 96.95%, τ^2^ = 0.15, *Q*(12) = 527.70, *P *< .0001). Another sensitivity analysis of studies found to be low risk of bias revealed a pooled OR of 2.20 (95% CI, 1.81-2.69) with considerable heterogeneity (*I*^2^ = 81.52%, τ^2^ = 0.04, *Q*(4) = 15.09, *P* = .0045) (Appendix B, Figure S3). Across individual studies, ORs ranged from 1.05 to 3.92.

**Figure 3:**
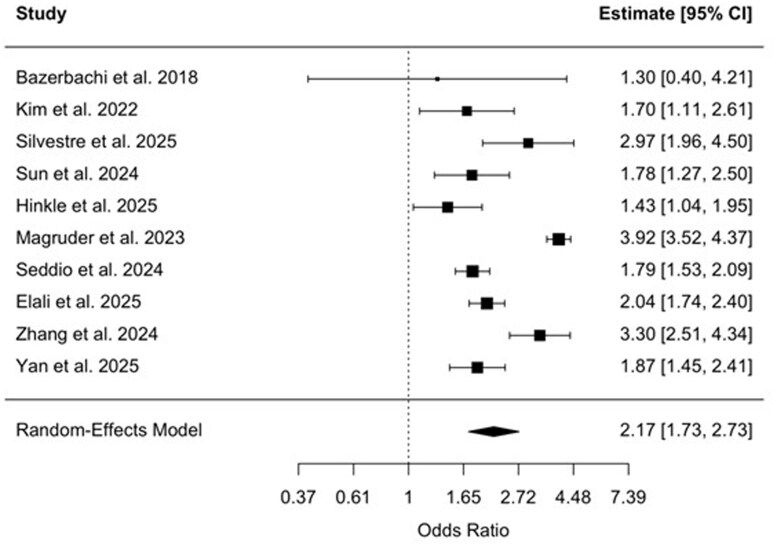
Forest plot of studies assessing the odds of acute kidney injury among patients with inflammatory bowel disease undergoing surgery. The pooled odds ratio was 2.17 (95% CI, 1.73-2.73), based on a random-effects model.

### Pooled ORs—surgical subgroup

**Figure 4:**
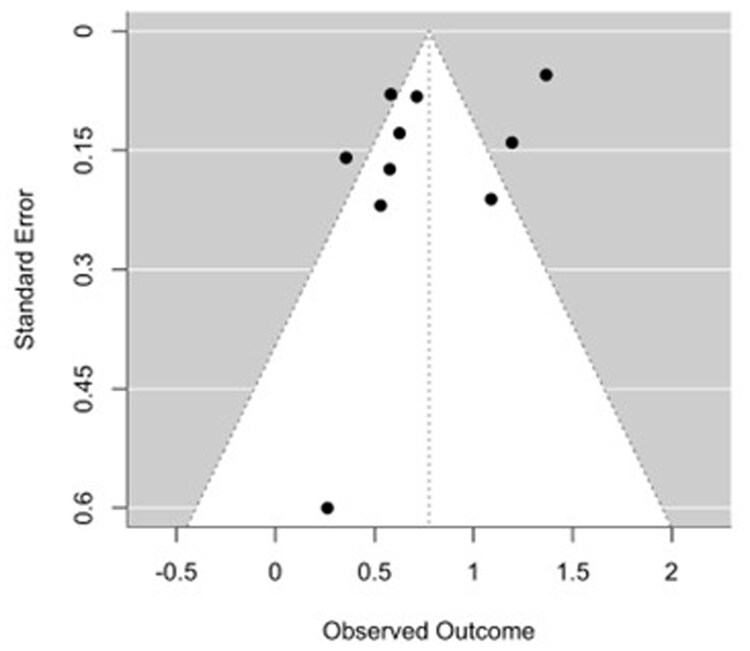
Funnel plot of studies evaluating the odds of acute kidney injury among surgical patients with inflammatory bowel disease. Egger’s test for funnel plot asymmetry was not significant (*P* = .259), indicating no major evidence of publication bias.

An analysis of 10 studies evaluating surgical patients demonstrated a significantly increased risk of AKI among individuals with IBD.^20–24^^,^^27–31^ The pooled OR was 2.17 (95% CI, 1.73-2.73, *P *< .0001) using a random-effects model (Figure 3). Between-study heterogeneity was considerable (*I*^2^ = 88.8%, τ^2^ = 0.1062), with a highly significant heterogeneity test (*Q*(9) = 119.73, *P *< .0001). Funnel plot asymmetry was not observed, and Egger’s test was nonsignificant (z = −1.13, *P* = .259) (Figure 4). Study-level ORs ranged from 1.30 to 3.92.

### Pooled ORs—orthopedic surgery subgroup

**Figure 5:**
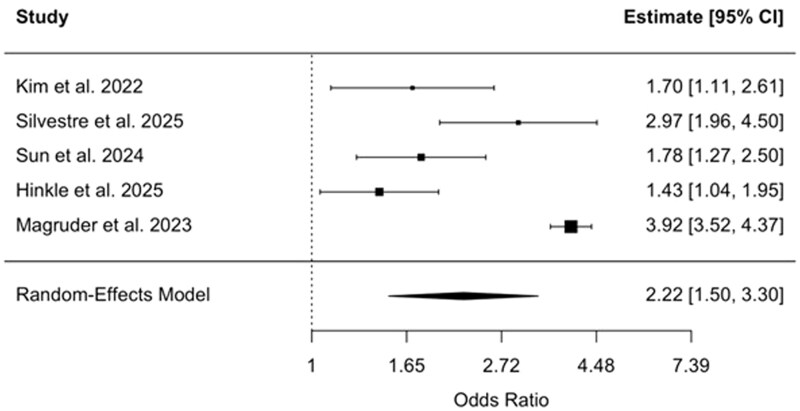
Forest plot of studies evaluating the odds of acute kidney injury among patients with inflammatory bowel disease undergoing orthopedic surgery. The pooled odds ratio was 2.22 (95% CI, 1.50-3.30), based on a random-effects model.

An analysis of 5 studies focusing on patients undergoing orthopedic surgery demonstrated a significantly increased risk of AKI among individuals with IBD.^27–31^ The pooled OR was 2.22 (95% CI, 1.50-3.30, *P *< .0001) using a random-effects model (Figure 5). Between-study heterogeneity was considerable (*I*^2^ = 89.3%, τ^2^ = 0.1732), and the heterogeneity test was statistically significant (*Q*(4) = 59.01, *P *< .0001). Individual study estimates ranged from ORs of 1.43 to 3.92.

### Pooled ORs—spinal surgery subgroup

**Figure 6:**
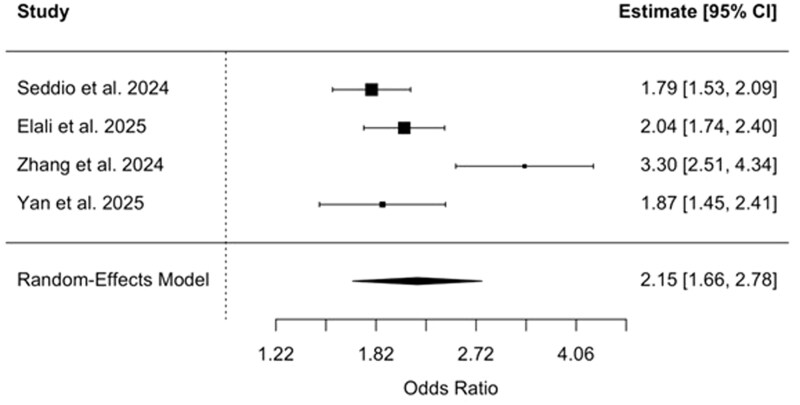
Forest plot of studies evaluating the odds of acute kidney injury among patients with inflammatory bowel disease undergoing spinal surgery. The pooled odds ratio was 2.15 (95% CI, 1.66-2.78), based on a random-effects model.

In 4 studies examining spinal surgery populations, patients with IBD had significantly higher odds of developing AKI compared to their non-IBD counterparts.^21–24^ The pooled OR was 2.15 (95% CI, 1.66-2.78, *P *< .0001) based on a random-effects model (Figure 6). Heterogeneity was considerable (*I*^2^ = 84.8%, τ^2^ = 0.0577), and the test for heterogeneity was statistically significant (*Q*(3) = 14.90, *P* = .0019). ORs ranged from 1.79 to 3.30.

**Figure 7:**
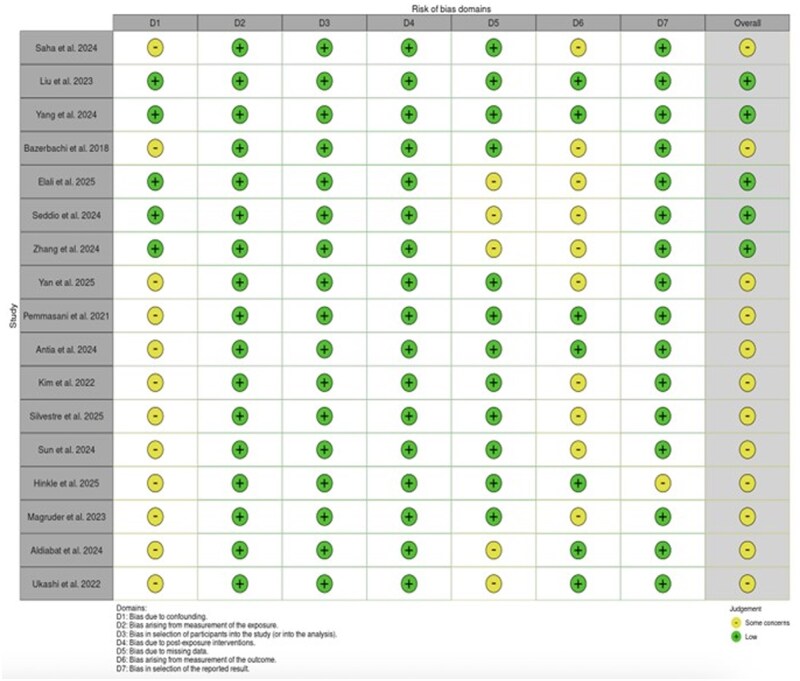
ROBINS-E traffic light plot.

### Pooled ORs—acute coronary syndrome subgroup

A meta-analysis of 2 studies evaluating patients with acute coronary syndrome demonstrated a modest but statistically ­significant association between IBD and the risk of AKI.^25^^,^^26^ The pooled OR was 1.06 (95% CI, 1.02-1.09, *P* = .0032), indicating an increased risk of AKI among patients with IBD for acute coronary syndrome. There was no evidence of between-study heterogeneity (*I*^2^ = 0%, τ^2^ = 0), and the heterogeneity test was not significant (*Q*(1) = 0.04, *P* = .842).

### Pooled ORs—infection subgroup

In a subgroup analysis of 2 studies focused on patients hospitalized for infections, the association between IBD and AKI did not reach statistical significance.^18^^,^^32^ The pooled OR was 2.00 (95% CI, 0.78-5.14, *P* = .148) using a random-effects model. Considerable heterogeneity was present (*I*^2^ = 92.1%, τ^2^ = 0.428), with a statistically significant heterogeneity test (*Q*(1) = 12.65, *P* = .0004).

### Risk of bias assessment

The ROBINS-E tool was used to evaluate the risk of bias in the included studies. None of the studies was rated as having a critical or high overall risk of bias (Figure 7). Most studies had some concerns, mainly in domains related to confounding (D1), missing data (D5), and outcome measurement (D6). Only 5 studies were considered to have a low overall risk of bias, which was due to strong adjustment for confounders, appropriate selection and measurement methods, and minimal missing data. Common limitations included not adjusting for key clinical factors such as baseline renal function, IBD severity, medication exposures, and comorbidities. Furthermore, many studies relied on International Classification of Diseases codes to define AKI, which offer high specificity but low sensitivity, thereby increasing the risk of misclassification and underreporting of events.

## Discussion

This systematic review and meta-analysis showed a statistically significant higher risk of AKI among hospitalized patients with IBD compared to those without IBD controls. Across 17 retrospective cohort studies involving over 20 million patients, the pooled OR for AKI was 1.87 (95% CI, 1.53-2.29). These findings quantitatively affirm prior large cohort studies reporting elevated adjusted risks of AKI in IBD^6^^,^^10^^,^^11^ and provide a meta-analytic synthesis of this risk in hospitalized IBD patients.

The risk of AKI was particularly elevated in surgical settings. IBD was associated with more than double the odds of developing AKI compared to non-IBD controls in the surgical context (pooled OR: 2.17, 95% CI, 1.73-2.73). This heightened risk was consistent across specific surgical subgroups, including orthopedic surgery (pooled OR: 2.22, 95% CI, 1.50-3.30) and spinal surgery (pooled OR: 2.15, 95% CI, 1.66-2.78). These findings reinforce prior ­observational studies that have linked IBD to worse surgical ­outcomes,^20–23^ although few have focused explicitly on renal complications. Notably, while previous studies have often centered on infectious or wound-related complications following surgery in IBD populations,^20–25^ current results suggest that renal complications may also arise.

In contrast, nonsurgical populations showed more variable findings. Overall, the nonsurgical subgroup as a whole did not demonstrate a statistically significant association with AKI (pooled OR: 1.41, 95% CI, 0.88-2.26). This is consistent with studies that have observed subclinical renal impairment or transient AKI episodes in outpatient or stable hospitalized IBD patients, which may also be underrecognized due to lack of routine monitoring.^7^^,^^10^^,^^33^^,^^34^

Among patients hospitalized with acute coronary syndrome, the pooled OR was 1.06 (95% CI, 1.02-1.09), reflecting a statistically significant increase in AKI risk. This is consistent with evidence that systemic inflammation in IBD can impair renal perfusion and increase susceptibility to hemodynamic or contrast-related kidney injury during cardiovascular admissions.^35–38^ Importantly, the ­increased risk of AKI extended even to admissions unrelated to intestinal disease activity, including ACS and orthopedic or spinal surgery. This suggests that IBD confers a baseline physiological ­vulnerability independent of active flares. Potential mechanisms include chronic low-grade systemic inflammation, impaired renal autoregulation, dysbiosis-related metabolic changes, and long-term exposure to nephrotoxic medications, all of which may reduce renal reserve even when IBD is clinically quiescent.^10^ Additionally, IBD patients frequently have nutritional deficiencies, altered fluid handling, altered gut-kidney axis signaling, and higher comorbidity burdens, which may render them less resilient during hemodynamic stressors such as anesthesia, major surgery, contrast exposure, or systemic infection.^11^ These mechanisms may explain why AKI risk persists across diverse hospitalization types and is not solely tied to disease flares or gastrointestinal pathology.

In the general inpatient population subgroup, the pooled OR was 1.79 (95% CI, 1.22-2.63), reinforcing that hospitalization itself may represent a period of elevated renal risk for patients with IBD, regardless of specific indications. This is in line with previous registry-based studies reporting increased AKI incidence during IBD-related hospitalizations, particularly in those with active disease.^6^^,^^9^^,^^35^ The infection subgroup, however, yielded a pooled OR of 2.00 (95% CI, 0.78-5.14), which was not statistically significant and showed considerable heterogeneity. The imprecision in this subgroup highlights the need for better data on the intersection of infection, inflammation, and renal injury in IBD. Across all analyses, heterogeneity was consistently high, possibly reflecting differences in patient populations, hospitalization indications, and database structures. Variability in case mix (eg, surgical vs. medical admissions), disease severity, comorbidity burden, and exposure to nephrotoxic medications may have also contributed. The combination of limited covariate data, heterogeneous study designs, and reliance on administrative codes for both exposure and outcomes introduces residual confounding and misclassification, which plausibly accounts for much of the between-study variability observed in our meta-analysis.

The nearly 2-fold increase in AKI risk among patients with IBD, especially the more than 2-fold rise in perioperative settings, underscores the potential need for systematic methods to assess and prevent kidney risk in this group. In surgical environments, perioperative care could extend beyond infection control and bleeding prevention to include proactive kidney protection strategies such as careful fluid management, cautious use of nephrotoxic drugs, and close postoperative monitoring of kidney function. In nonsurgical situations, where AKI might occur without symptoms or be temporary, routine checks of serum creatinine and urinalysis during disease flare-ups, hospital stays, or when initiating potentially nephrotoxic treatments (eg, 5-ASA, calcineurin inhibitors, biologics) can facilitate earlier detection and intervention.

Residual confounding remains an important consideration when interpreting these results. Most included studies did not provide granular data on IBD activity (eg, clinical indices, biomarkers, endoscopic scores), specific disease phenotypes, or detailed exposure to IBD therapies, and very few reported prior IBD-related surgeries beyond broad adjustment terms. For example, factors such as active inflammation, penetrating or extensive disease, and prior surgical interventions plausibly increase AKI risk in high-risk settings by amplifying hemodynamic instability, infection risk, and nephrotoxic exposures.^36^ Because these variables were incompletely measured and rarely explored in stratified analyses, our pooled estimates may partially reflect clustering of high-risk clinical scenarios among hospitalized IBD patients rather than the effect of an IBD diagnosis alone.

In addition, AKI is not unique to IBD, and contextualizing these findings alongside other chronic disease populations is important. Comparable or even greater increases in AKI risk are well documented in other chronic disease states. Diabetes mellitus, hypertension, and CKD disease substantially elevate the risk of AKI, with CKD conferring up to a 10-fold increase and lower baseline estimated glomerular filtration rate (eGFR) or elevated albuminuria markedly amplifying susceptibility.^37^^,^^38^ Therefore, while AKI is a recognized extraintestinal manifestation of IBD, the phenomenon is not disease-specific but rather reflects shared pathways of systemic inflammation, hemodynamic instability, medication exposures, and surgical or hospitalization-associated risk.

Limitations of our study include the exclusive reliance on retrospective cohort data, high heterogeneity in several analyses, and potential residual confounding due to incomplete adjustment for disease activity, medication use, and baseline kidney function. In addition, the surgical versus nonsurgical subgroup analysis was not prespecified in the PROSPERO-registered protocol and was introduced post hoc based on emerging heterogeneity and clinical relevance, so this deviation should be considered when interpreting subgroup findings. Additionally, varying definitions of AKI and a lack of granularity on IBD phenotype (eg, severity, disease activity) and potential nephrotoxic exposure limited subgroup depth.

## Conclusion

This systematic review and meta-analysis suggests a potential link between a diagnosis of IBD and AKI, particularly in surgical settings. While nonsurgical and inpatient populations also show increased risk, the findings are less consistent. Future studies should focus on improving risk assessment tools and testing these preventive strategies.

## Supplementary Material

gwag004_Supplementary_Data

## Data Availability

The underlying data and R statistical code will be made available on reasonable request to the corresponding author.
