# Supplementary material for: Risk of acute kidney injury in hospitalized patients with inflammatory bowel disease: a systematic review and meta-analysis
Source: J Can Assoc Gastroenterol. 2026 Feb 13;9(3):137–46. doi: 10.1093/jcag/gwag004 (PMC13232503; doi:10.1093/jcag/gwag004)
Supplement: gwag004_Supplementary_Data [file gwag004_supplementary_data.zip › gwag004_Supplementary_Data/Appendix_A_search_strategy.docx]

**APPENDIX A: Search Strategy (July 9, 2025)**

**MEDLINE <1946 – 2025> Search (380 results)**

1. inflammatory bowel disease.mp. or Inflammatory Bowel Diseases/ (75599 results)

2. ulcerative colitis.mp. or Colitis, Ulcerative/ (66648 results)

3, Crohn's disease.mp. or Crohn Disease/ (70037 results)

4. acute kidney injury.mp. or Acute Kidney Injury/ (82076 results)

5. acute renal failure.mp. (26145 results)

6, Renal Dialysis/ or Dialysis/ or dialysis.mp. (200359 results)

7. 1 or 2 or 3 (150078 results)

8. 4 or 5 or 6 (279594 results)

9. 7 and 8 (380 results)

**Embase <1974 – 2025> Search Strategy (2010 results)**

1. inflammatory bowel disease.mp. or Inflammatory Bowel Diseases/ (141003 results)

2. ulcerative colitis.mp. or Colitis, Ulcerative/ (120843 results)

3, Crohn's disease.mp. or Crohn Disease/ (138253 results)

4. acute kidney injury.mp. or Acute Kidney Injury/ (162252 results)

5. acute renal failure.mp. (38107 results)

6, Renal Dialysis/ or Dialysis/ or dialysis.mp. (326581 results)

7. 1 or 2 or 3 (266771 results)

8. 4 or 5 or 6 (466807 results)

9. 7 and 8 (2010 results)

**Scopus (248 results)**

TITLE-ABS-KEY(("inflammatory bowel disease" OR "ulcerative colitis" OR "Crohn's disease") AND ("acute kidney injury" OR "acute renal failure"))

**PubMed (355 results)**

((inflammatory bowel disease) OR (ulcerative colitis) OR (Crohn's disease)) AND ((acute kidney injury) OR (acute renal failure))

**Cochrane Central Register of Controlled Trials (CENTRAL) (29 results)**

1. inflammatory bowel disease.mp. or Inflammatory Bowel Diseases/ (4618 results)

2. ulcerative colitis.mp. or Colitis, Ulcerative/ (7082 results)

3, Crohn's disease.mp. or Crohn Disease/ (5900 results)

4. acute kidney injury.mp. or Acute Kidney Injury/ (5653 results)

5. acute renal failure.mp. (1526 results)

6, Renal Dialysis/ or Dialysis/ or dialysis.mp. (18724 results)

7. 1 or 2 or 3 (13522 results)

8. 4 or 5 or 6 (24356 results)

9. 7 and 8 (29 results)
