# Supplementary material for: Risk of acute kidney injury in hospitalized patients with inflammatory bowel disease: a systematic review and meta-analysis
Source: J Can Assoc Gastroenterol. 2026 Feb 13;9(3):137–46. doi: 10.1093/jcag/gwag004 (PMC13232503; doi:10.1093/jcag/gwag004)
Supplement: gwag004_Supplementary_Data [file gwag004_supplementary_data.zip › gwag004_Supplementary_Data/Appendix_B_supplemental_figures_tables.docx]

Appendix B: Supplemental Figures


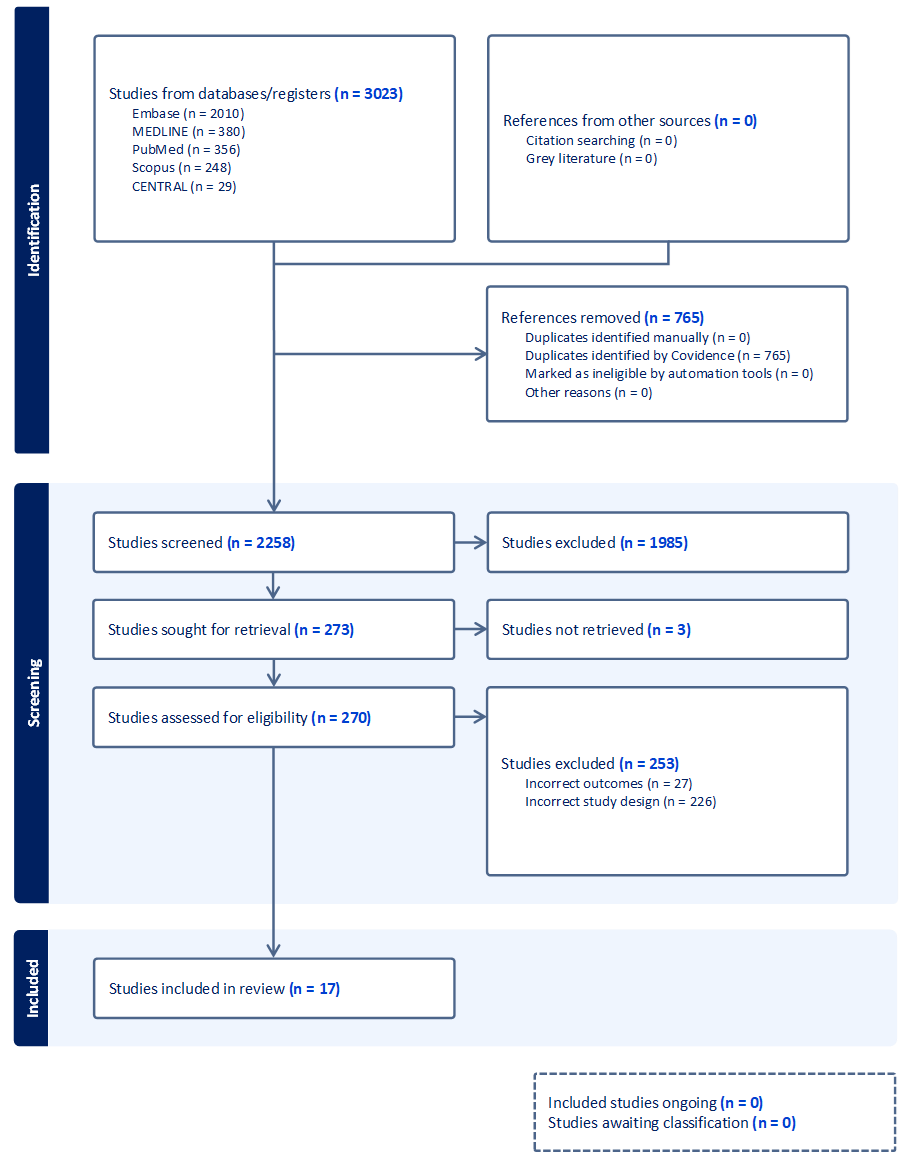


***Supplemental Figure 1.*** *Summary of the search results and study selection in a PRISMA flow diagram.*


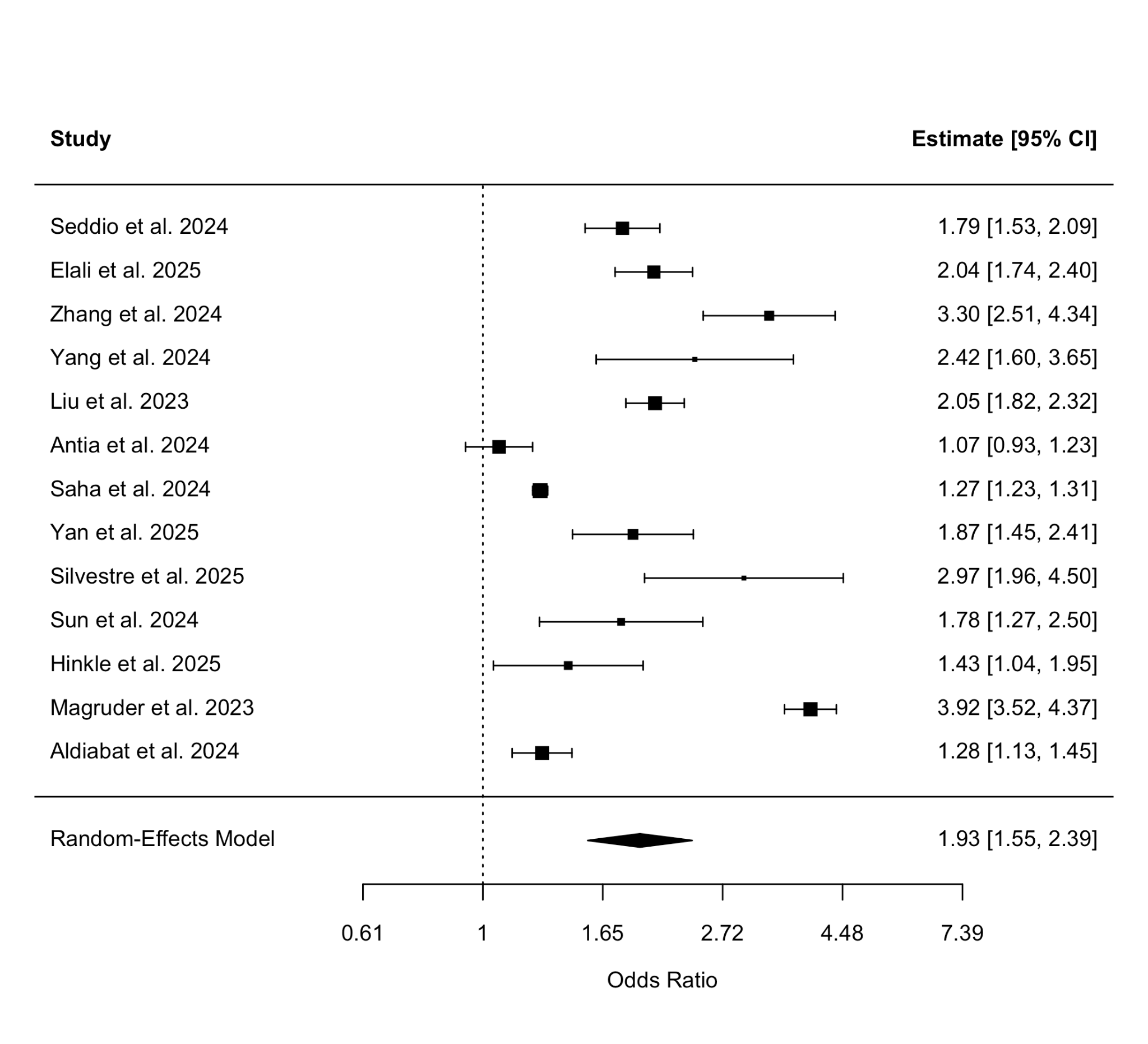
***Supplemental Figure 2.*** *Forest plot of 13 studies published between 2023 and 2025 assessing the odds of acute kidney injury in patients with inflammatory bowel disease. The pooled odds ratio was 1.93 (95% CI: 1.55-2.39) using a random-effects model.*


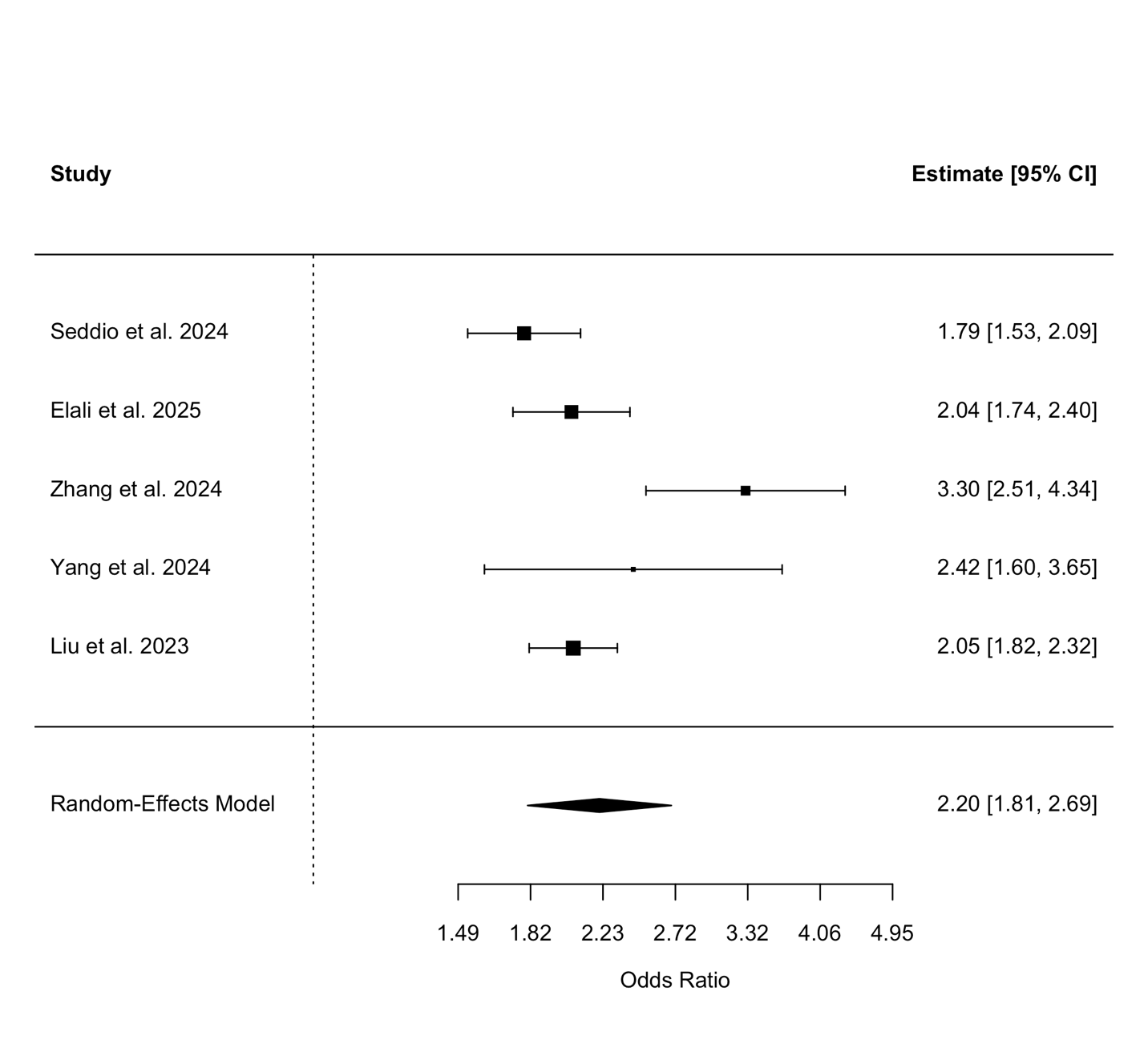


***Supplemental Figure 3.*** *Forest plot of 5 studies judged to be at a low risk of bias assessing the odds of acute kidney injury in patients with inflammatory bowel disease. The pooled odds ratio was 2.20 (95% CI: 1.81-2.69) using a random-effects model.*
